# Commencements

**Published:** 1897-07

**Authors:** 


### COMMENCEMENTS.

### University of Buffalo—Dental Department.

The Dental Department of the University of Buffalo held its fifth annual commencement exercises, in connection with the Departments of Medicine and Pharmacy in Music Hall, Buffalo, on the evening of April 27th, when the Chancellor conferred upon the following named students the Degree of Doctor of Dental Surgery.

The whole number of students matriculated for the term was 222. The number of Seniors was 70. The number of Junior class numbered 70. The Freshmen numbered 82.

Bertram John Baker
Harry Eddy Barnhart
Jarvis Barraclough
Frederic S Belding
Alfred Milton Borten
Warren Joseph Boyd
William Penn Brinton
Alfred Byron Cairnes
Colin Cameron
Harry Moore Clark
Alfred Rayson Cook
Charles Stephen Deuel
Jay Herbert Dower
James Vernon Faulkner
John Vosburgh Flaherty
Ferdinand John Freitag
Frank Arthur Gough
Francis L Greene Ph B
Frederick Dodge Greene
Beverly Chew Guile
Leverett L Hamilton
George Tyler Head M D
Perry Frederick Hepp.
George Taylor Hickelton
Abram Lauder Hipwell
Carey Elijah Janes
Henry H Ketcham
Van Albert Lacy
William Charles Luetti
George Thomas Lord
Harry Benjamin Lyon
Arthur Bastedo Magee
William Hyde Marks
Charles Jason McClure
William S McFarlane
John Frederick McPhee
Frank Chace McPherson
Henry Stuart Merkley
William Drysdale Moss
Theodore Perkins Moyer
Robert Muir
Frederic Bryant Niles
Leonard Earl Noble
Henry Richard O'Brien
Seth Theodore Paine
Charles Bicknell Payne
Charles Henry Plumstead
William Thomas Potter
Harry Pond Rawson
Louis St George Rickards
Wilbur Salathiel Rose
Samuel Rowan
Clarence Levi Roys Ph B
Rolla Sabine
Daniel George Simmonds
Frank Walton Smith
Howard Eugene Stone
John Marcus Tanzer
Harry Vedder Tefft
Anna Mathilde Thrane
Frank Hicks Underwood
Clarence R Waldron
Charles Henry Whitlock
Harry Lawson Whipple
Melrose Williams Wright
Edward Arthur Wood
George Daw Wood

### Missouri Dental College.

The thirty-first annual commencement of the Missouri Dental College, Dental Department of Washington University, was held at the Germania Theater, (14th St. Theater), on Thursday evening, April 29th, in connection with that of the St. Louis Medical College. The address to the class was by Dr. Morris K. Levi, of New York. The degree of D. M. D. was conferred by Chancellor Chaplin of the University. Faculty prize awarded to Dr. Louis W. Bartel; S. S. White prize to Samuel Williams; John

Rowan D. M. Co. prize to Otto J. Fruth; St. Louis D. M. Co. prize to Rudolph Widman; honorable mention to Henry T. Clark, John H. Drown and William H. Evans. Matriculates, one hundred and five. Graduates, twenty.

| NAME. | RESIDENCE. | NAME. | RESIDENCE. |
|---|---|---|---|
| Louis W. Bartel | Illinois | Paul W. Lenze | Missouri |
| William Brown | Illinois | Hugo A. Lehmberg | Missouri |
| Oscar V. Carrell | Iowa | Thomas C. Miller | Missouri |
| Henry T. Clark | Missouri | Leslie E. Rawson | Missouri |
| Homer M. Cook | Iowa | Aaron Rothschild | Kansas |
| John H. Drown, M. D | Missouri | Charles E. W. Sommers | Missouri |
| William H. Evans | California | Albert P. Tschirner | Missouri |
| Otto J. Fruth | Missouri | Rudolph Widman | Missouri |
| Montgomery M. Fitzgerald | Missouri | Samuel Williams | Missouri |
| Hunter S. Hyndman | Illinois | George W. Yeargain | Missouri |

### Birmingham Dental College.

The commencement exercises of the Birmingham Dental College were held (in connection with the Birmingham Medical College) in O'Brien's Opera House, Birmingham, Ala., on Tuesday evening, April 7th, 1897.

The address to the graduating class was delivered by General Fred. S. Ferguson of Birmingham. The number of matriculates for the session was forty (40). The degree of D. D. S. was conferred on the following graduates by T. M. Allen, D. D. S., Dean:

| NAME. | RESIDENCE. | NAME. | RESIDENCE. |
|---|---|---|---|
| C. E. Coleman | Georgia | John Goodwin | Alabama |
| D. F. Florence | Alabama | J. P. Haley | Alabama |
| H. S. Florence | Alabama | R. P. McTyeire | Alabama |
| J. A. Freeman | Alabama | | |

### Department of Dental Surgery of the Detroit College of Medicine.

The fourth annual commencement exercises of the Department of Dental Surgery of the Detroit College of Medicine, took place at Harmonie Hall, Thursday evening June 10th, 1897, before a large and cultured audience.

Schremser's orchestra opened the proceedings, and at intervals rendered some beautiful selections. Rev. Morgan Wood led in prayer.

Prof. G. L. Field gave a farewell address to the graduating class. In the absence of Hon. Sydney D. Miller, Prof. E. L. Shurly, vice-president of the college, conferred the degrees. Later on he delivered an able address. Dr. E. C. Skinner presented the Skinner gold medal for general proficiency, the recipient being Norman B. Fox, D. D. S. A silver medal was awarded by the faculty to John S. Hall, D. D. S., so close was the competition between him and Mr. Fox. M. E. Stafford, D. D. S., the class prophet, in a clever poem foretold the future of his recent classmates. Twenty-four candidates received the degree of Doctor of Dental Surgery.

| NAME. | RESIDENCE. | NAME. | RESIDENCE. |
|---|---|---|---|
| A. H. Bretz | Michigan | W. H. Martmer | Michigan |
| W. A. Brown | Ontario | C. C. Noble | Michigan |
| M. A. Banks | Illinois | W. R. Rorke | Ontario |
| H. A. Currie | Ontario | A. Rheiner | Michigan |
| F. Carrothers | Ontario | W. E. Sanborn | Michigan |
| D. L. Duncan | Michigan | E. B. Stafford | Michigan |
| J. A. Dorr | Michigan | M. E. Stafford | Michigan |
| N. B. Fox | Ontario | F. Vail | Michigan |
| E. Frumveller | Michigan | G. L. Van Luven | Ontario |
| J. S. Hall | Ontario | H. V. Jackson, M. D. | Michigan |
| D. A. MacMillan | Ontario | E. W. Oswald, M. D. | Michigan |
| T. J. McDowell | Ontario | G. Webster | Ontario |

### Western Reserve University—Dental College.

The annual commencement of Western Reserve University--Dental College, was held May 17th, 1897, at Association Hall, Cleveland, Ohio. Rev. Livingston Jalor, D. D., delivered an address entitled "Home Thrusts." The degree of Doctor of Dental Surgery was conferred by the President Charles F. Thwing, D. D., upon the graduates. Number of students, eighty-six attending sessions; graduates, thirty-one, as follows:

| | | |
|---|---|---|
| Fred. Sampson Anderson | Charles Andrew Fink | John Augustus Osborne |
| Lyman S. Armstrong | Ellason Francis Grose | Reuben Wilson Parker |
| Clayton Royal Baldwin | Arthur LaFayette Higgins | Aubrey Leard Parsons |
| Morley James Beal | William C. Honeywell | M. Curtis Ramaley |
| William Livingston Beal | Harry Milton King, A. B. | William Folwell Spargar |
| Carl Foster Blair | Fred. Leslie Ludwick | William Jacob Stephan |
| William Dudley Bolton | William M. Megginson | Walter Paul Smith |
| Luther L. Bosworth | Frederick LaValle Miles | Burt Edward Saunders |
| Clarence Wilson Davis | Frank Aloy Moran | Charles F. Wallace |
| William Rodell Dixon | Martin Henry Morrison | Frank Levy West |
| Dexter Henry Fairbanks | | |

### College of Dentistry—University of Minnesota.

The commencement exercises of the College of Dentistry, University of Minnesota, were held in University Armory, in connection with other schools of the University. The degree of Doctor of Dental Surgery was conferred by the President of the University, Cyrus Northrop, L. L. D., upon the following persons, viz:

| NAME. | RESIDENCE. | NAME. | RESIDENCE. |
|---|---|---|---|
| E. R. Annis | Minnesota | J. M. Hall | Minnesota |
| W. W. Baker | Minnesota | B. A. Herrick | Wisconsin |
| E. E. Buell | Minnesota | C. A. Leonard | Wisconsin |
| T. F. Cooke | Minnesota | W. McCadden | Minnesota |
| Miss M. J. Covey | Minnesota | Mrs. E. P. Medary | Iowa |
| H. B. Denton | Minnesota | W. A. Moore | Minnesota |
| W. T. Evans | Indiana | T. A. Pattison | Minnesota |
| J. L. Frederick | Minnesota | H. A. Pullen | Minneseta |
| J. W. S. Gallagher | Minnesota | S. A. Sanderson | Minnesota |
| H. B. Godfrey | Minnesota | J. W. Shankland | Iowa |
| H. S. Godfrey | Minnesota | E. Shumpik | Minnesota |
| M. S. Goodnow | Minnesota | T. Spence | Illinois |
| T. P. Hagerty | Minnesota | F. L. Stephan | Minnesota |
| A. E. Hawkinson | Minnesota | B. T. Stephens | Minnesota |

### University College of Medicine—Dental Department, Richmond, Va.

The commencement exercises of the Dental Department of the University College of Medicine, of Richmond, Va., were held in the Academy of Music in that city, on the night of April 29, 1897.

The address before the graduating class was delivered by Hon. Walter Clark, of Raleigh, N. C., Associate Justice of the North Carolina Supreme Court, and was a masterly production, adding distinction to his already great reputation as a scholar and orator.

The following gentlemen were awarded the degree of D. D. S.:

| NAME. | RESIDENCE. | NAME | RESIDENCE. |
|---|---|---|---|
| Herbert W. Barksdale | Virginia | James P. Keech | North Carolina |
| L. L. Dameron, Jr. | North Carolina | W. H. O. McGehee | Virginia |
| Ernest M. Hardy | England | William D. Willis | Virginia |
| T. D. Hubbard | Virginia | | |

Prizes were awarded as follows: Faculty Medal, for highest average on all studies for the whole three year's course, W. D. Willis. Cowardin Prize, best essay on diseased pulps, H. W. Barksdale. C. L. Steel Prize, best specimen of bridge work, H. W. Barksdale. Grant Prize, best gold filling, T. D. Hubbard.

The exercises closed with a banquet to the graduates and alumni at the Hotel Jefferson.

### Dental Department, Southern Medical College, Atlanta, Ga.

The tenth annual commencement exercises of the Dental Department of the Southern Medical College, was held in the Grand Opera House, Atlanta, Ga., March 23d, 1897.

The degree of Doctor of Dental Surgery was conferred upon nineteen applicants, as follows:

| NAME. | RESIDENCE. | NAME. | RESIDENCE. |
|---|---|---|---|
| J. L. Adams | Georgia | S. W. Harris | Georgia |
| E. R. Baker | Georgia | R. L. Hunter | Georgia |
| R. W. Blackstone | Wisconsin | H. N. Gallagher | Georgia |
| D. D. Beekman | Florida | M. W. Gage | Mississippi |
| F. E. Clark | California | Chas. W. Lokey | Alabama |
| G. W. Collins | Georgia | J. A. Martin | Louisiana |
| J. H. F. Eagans | South Carolina | A. L. McArthur | Georgia |
| D. L. Farmer | Georgia | Wip Robinson | Tennessee |
| J. H. Foster | Alabama | J. W. Robinson | Maine |
| Otis Gilbert | Georgia | | |

The annual oration was delivered by the Hon. Albert H. Cox, of Atlanta. Valedictory address by J. H. Foster of Alabama.

### Northwestern College of Dental Surgery.

The commencement exercises of the Northwestern College of Dental Surgery were held at Kimball Hall, Monday, April 5th, at 2:30 p. m., with the following programme: Invocation, Rev. H. R. Neeley; Salutatorion, Harry A. B. McConnell; Faculty Address, Prof. F. A. Hodson, M. D.; Conferring of Degree, Prof. W. X. Sudduth, M. D., D. D. S.; Valedictorian, William E. Herbert; Address by the Dean, L. L. Davis, D. D. S.

The degree of Doctor of Dental Surgery was conferred upon the following graduates:

| | | |
|---|---|---|
| H. A. McConnell | M. J. Dougherty | H. J. Tarr |
| W. E. Herbert | C. W. Hoppman | O. B. Hayden |
| P. J. McCarthy | E. LaFrienier | C. F. Sinclair |
| F. A. Winslow | C. E. Winslow | St. L. Estes |
| J. M. Anderson | F. E. Russell | John Davern |
| J. F. Heintz | | |

### University of Maryland—Dental Department.

The annual commencement of the University of Maryland, Dental Department, was held in the Lyceum Theatre, Wednesday evening, March 31st, 1897, at 8 o'clock. Address to the graduates was given by Rev. M. B. Wharton, D. D. The Class oration was delivered by N. C. Raoul. Prizes were awarded by Prof. Francis T. Mills, as follows: two University gold medals for highest grade, M. N. King and D. E. McConnell; honorable mention for second highest grade, F. R. Thomas; S. S. White prize, F. B. Weller; Snowden & Cowman Manufacturing Co. prize, F. S. Anderson; Prof. Harris' prize, P. Russell; Prof. Gorgas' prize, J. W. Faucette; Dr. C. J. Grieves' prize for bridge-work, R. L. Henry; Dr. C. J. Grieves' prize for senior crown work, W. G. Boyd.

The degree of D. D. S. was conferred by the Hon. John P. Par, Acting Provost of the University, upon the following candidates:

| NAME. | RESIDENCE. |
|---|---|
| Albert A. Aiken | Texas |
| Fred. S. Anderson | New Brunswick |
| Edgar Jarratt Applewhite | Virginia |
| Charles E. Ayres | Pennsylvania |
| Albert D. Baker | Maryland |
| J. Henry Besore | Maryland |
| Samuel D. Bodine | Pennsylvania |
| Walter G. Boyd | Maryland |
| H. C. Raoul Breault | Connecticut |
| John H. Bushong | Virginia |
| E. A. Charbonnel | Washington State |
| Clarence E. Collins | Maryland |
| James L. Duff | Canada |
| Arthur Paul Farley | Connecticut |
| John Wm. Faucette | North Carolina |
| Eugene Finnegan | New York |
| Newton Fernando Foote | New York |
| John McClosky Foreman | Pennsylvania |
| A. Hutchings Frith | Bermuda |
| John Gardner | Maryland |
| Arthur Perry Gordy | Georgia |
| W. Gordon Gould | Connecticut |
| Curtis LeRoy Hartman | Pennsylvania |
| Charles F. Heisler | Pennsylvania |
| Robert Lee Henry | Georgia |
| John Griffin Hoffman | Virginia |
| Marion N. King | Virginia |
| John M. MacDonald | New Zealand |
| Daniel T. Maloney | Connecticut |
| Chesley Martin | Virginia |
| Daniel E. McConnell | South Carolina |
| C. Howard McNutt | Nova Scotia |
| Robert W. Morton | Pennsylvania |
| Wirt H. Moseley | Virginia |
| Abram V. Mowel | New York |
| R. Murray Muir | Canada |
| Robert Cassell Nicodemus | Maryland |
| Robert Gordon Norfleet | Virginia |
| Samuel P. Norris | North Carolina |
| Henry Ignatius O'Connor | Georgia |
| Henry Newton Pheneger | Pennsylvania |
| Thomas Lloyd Posey | Kentucky |
| R. Russell Reiff | Pennsylvania |
| James Leftwich Rogers | Tennessee |
| Frank Sidney Robinson | Florida |
| Louis H. Russell | South Carolina |
| Percy Russell | Maryland |
| William Close Seaton, Jr. | New York |
| Walter Owens Stack | Delaware |
| J. Russell Steele | Pennsylvania |
| Stanley Steele | Maryland |
| Max Stein | Russia |
| Charles C. Switzer | New York |
| Elisha S. Taylor | Maryland |
| Fred. Rudolf Thomas | Nova Scotia |
| Alexander Tribble | Missouri |
| Patrick J. Vaughan | Georgia |
| J. Somes Watts | Canada |
| Julius Weinberger | New York |
| Franklin Babbitt Weller | New York |
| Alpha Ayer Williams, Jr. | Georgia |
| Samuel B. Wrightson | Maryland |

### University of California College of Dentistry.

The commencement exercises of this institution were held at Odd Fellows' Hall, San Francisco, Cal., Wednesday evening, June 16th, 1897, at 8 o'clock.

Rev. W. W. Bolton offered prayer, after which came an address in behalf of the faculty, by A. H. Suggett, B. S., D. D. S., after which the degree of D. D. S. was conferred by Martin Kellogg, A. M., L. L. D., president of the University, upon the following candidates :

Julian Woods Ashley
Benjamin Avery Bosqui
Edwin Andrews Clay
Charles Alfred Coffin
Orville Mirtland Colburn
Asa Weston Collins
Medora Vaux Croall
Stephen Russell Cushing
George Samuel Donnelley
Judge Haley Durham
Edward Sewell Fiske
Howard West French
Ray Edson Gilson
Herman Pierce Hanson
Frederic Wm. Harnden
William M. Herrington
Theodore Shelton Higgins
William Robin Holladay
Walter Renwick Hughes
Charles F. Howard Jarvis
Frank D. Johnson
Paul Clinton Jones
Edmund Douglas Keeffe
Louis Joseph Kerwin
Clair Cutting Marckres
Charles Joseph McCarthy
Milton McMurry
Julius Ira Morris
James Arthur Plunkett
Thomas Hopkins Quirk
George W. Raymond
Harry C. Reynolds
Frank Oniska Robinson
Helen May Rulison
Edward Weld Russell
Fannie Elsie Scott
Edwin Matthew Stealey
Henry Stiles
Charles Maurice Sumner
George Henry Tomkins
Edward Albright Upton
Martin Wachs
Thomas Rattray Wheeler

### Ohio College of Dental Surgery.

The fifty-first annual commencement of the Ohio College of Dental Surgery, department of Dentistry-University of Cincinnati, was held at the Odeon, Tuesday, April 6th, 1897, at 8 o'clock P. M., and the following programme was gone through with : Invocation, conferring of degrees, Dr. Frank A. Hunter, President of the Board of Trustees; Awarding of Prizes and Remarks by the Dean, Prof. H. A. Smith; Address, Rev. Warren G. Partridge; Class Address, Shotwell H. Roff, Cincinnati, Ohio.

The following candidates had the degree of D. D. S. conferred upon them :

| NAME. | RESIDENCE. |
|---|---|
| Edward Arthur Baldwin | Ohio |
| Elmore Franklin Barrington | Ohio |
| Charles Chase Boggess | Ohio |
| John Andrew Bouvy | Kansas |
| Harry Clay Bowen | Ohio |
| Harry B. Buchanan | Ohio |
| Norwood DeVacht Chamberlin | Ohio |
| Philip Sheridan Chesnut | Ohio |
| Hugh Robert Conklin | Ohio |
| Burdette Lee Conway | Ohio |
| Frank Austin Dille | Colorado |
| Clinton Howard Doss. | Illinois |
| Ralph William Ernest | Ohio |
| Clifford Thomas Eskey | Pennsylvania |
| William Thomas Embree | Missouri |
| George Fischer | Pennsylvania |
| Homer Curtis Foster | Ohio |
| John Jay Foster | Nebraska |
| Mrs. Nellie E. Freeman | Indiana |
| Edgar E. Heizer | Ohio |
| Emil Louis Haring | Indiana |
| Thomas Alonzo Hyer | Ohio |
| John Frank Herdliska | Ohio |
| Warren B. Harris | Indiana |
| Lewis Thatcher Ivins | Ohio |
| Claude Everett Keeney | West Virginia |
| Jottie Hopper Kendall | Ohio |
| William O. Kuechler | Pennsylvania |
| John Henry Marvin | Pennsylvania |
| Alviere Milligan | Ohio |
| James Maxwell Monfort | Ohio |
| Orlando Clinton Moon | Ohio |
| Miss Ella E. McKallip | Pennsylvania |
| Milton Grant Phillips | Ohio |
| James Thomas Ralston | Kentucky |
| Forster Robinson | Pennsylvania |
| Will Eckman Robinson | Ohio |
| Shotwell H. Roff | Ohio |
| Albert Richardson Riggs | Ohio |
| August Schwartz | Ohio |
| Joseph C. Sheets. | Ohio |
| Albert Howard Sherick | Ohio |
| Harry Armstrong Smith | Ohio |
| David Stern | Ohio |
| Herbert Hoffman Truesdell | Ohio |
| John Wallace Taylor | Kentucky |
| Duncan Pendleton Turner | Ohio |
| Frank Reynolds VanMetor | Ohio |
| Frank Moulton Warwick | Ohio |
| Benjamin James Wolf | Ohio |

Matriculates, 212. Graduates, 50.

### Kansas City Dental College.

The fifteenth annual commencement of this institution was held at Coates Opera House, at Kansas City, Wednesday evening, March 31st, 1897.

Invocation by the Rev. W. P. George. Address on behalf of graduating class, Claude Backus. Address by Dr. G. Newkirk of Chicago. Faculty address by Walter S. Wheeler, M. D.

The degree of D. D. S. was conferred by Dr. A. H. Thompson, President of the Faculty, upon the following graduates:

| NAME. | RESIDENCE. |
|---|---|
| Arnelle Delbert Andrews | Iowa |
| Claude Backus | Illinois |
| Guy Collins Colman | Missouri |
| William Emery Cutler | Kansas |
| Wesley Roberts Crawford | Kansas |
| Charles William Chalfant | California |
| Freeman Davis | Illinois |
| James Pleasant Dawson | Missouri |
| Howard Stanton Engle | Iowa |
| Harry William Fessenden | Kansas |
| Roger C. Fields | Missouri |
| James Hamilton Graham | Texas |
| Mark Dryden Gwinn | Missouri |
| Clinton Thomas Hart | Kansas |
| Charles LaFafette Hopkins | Missouri |
| A. Porter Hults | Kansas |
| Shelley Keats Johnson | Iowa |
| James Thomas Keith | Kansas |
| George Henry Kittell | Kansas |
| Leonard D. Koger | Missouri |
| Thaddeus S. Kelly | Missouri |
| Curtis Lee Larmer | Missouri |
| Frank Edward Leviston | California |
| Cullen Bryant McCarty | Iowa |
| William Asa Moore | Missouri |
| William H. Nipps | Missouri |
| John William Netherton | Missouri |
| Simon Guy Numbers | Kansas |
| Robert Henry Pendleton | Missouri |
| Thomas Edward Purcell | Kansas |
| Clyde M. Pancoast | Nebraska |
| John Armstrong Reily | Missouri |
| Elby Drue Rogers | Kansas |
| George Ruddell, Jr | Kansas |
| Edward Holcomb Stiles, Jr. | Missouri |
| William Samuel Thos. Smith | Missouri |
| George Clark Seyster | Kansas |
| John Thomas Wilson | Missouri |
| Elijah Gwinn Winkler | Kansas |

### Marion Simms College of Medicine—Dental Department.

The annual commencement was held on the evening of April 10th, 1897, in the 14th Street Theatre.

Invocation by Rev. J. F. Cannon. Announcement by the dean, Dr. Y. H. Bond. Address, "The Medical Needs of Armenia and Syria," after which the degree of D. D. S. was conferred by the Dean, upon the following candidates:

| NAME. | RESIDENCE. |
|---|---|
| John Wesley Austin | Missouri |
| Walter Enos Brown | Illinois |
| William Easterbrook Barter | Indiana |
| Howard Elmer Church | Missouri |
| Thomas Wright Clark | Arkansas |
| Charles Bernard Coleman | Missouri |
| Thomas Prior Fristoe | Missouri |
| Joseph Boyd Gordon | Missouri |
| Amos Fader Lewis | New Mexico |
| George Henry Parsons | Iowa |
| Henry Rodemich | Missouri |
| Thomas Henry Shekelton | Missouri |
| Zeph Tibbetts | Illinois |
| Rudolph Albert Vogt | Missouri |
| Gilbert Ridgway Williams | Indiana |

An address to the graduates was delivered by Rev. John Mathews. Benediction by Rev. Cannon.

LL.D., the faculty address by W. F. Kuhn, A.M., M.D., and the valedictory by Thomas W. Lyell, D.D.S.

The number of matriculates for the session was two hundred and twenty.

The degree of D.D.S. was conferred on the following graduates by D. J. McMillen, M.D., D.D.S., Dean of the Faculty:

| NAME. | RESIDENCE. | NAME. | RESIDENCE. |
|---|---|---|---|
| Herman F Branstetter | Missouri | William Dillingham Kirby | Kansas |
| William Broadbent | Missouri | Frank Gilkeson Lobban | Missouri |
| Edwin Sever Brown | Missouri | Thomas Walter Lyell | Missouri |
| Edgar Pearson Brown | Missouri | Francis Marion McDonald | Texas |
| Clarence Manfred Burris | Iowa | Thomas McMillan | Missouri |
| Willis Victor Chapin | Kansas | Welby Stanley Morrow | Iowa |
| Christopher C Clark | Missouri | Joseph Shelby Mirick | Missouri |
| John Logan Clark | Missouri | Ralph Hammond McCrum | Missouri |
| Russell Elmer Covey | Missouri | Omar Preston Muckley | Ohio |
| Edward Abner Dabbs | Missouri | Orin Knisley Muckley | Ohio |
| George Whitman Downing | Missouri | John A Parker | Missouri |
| Fannie Delaney | Missouri | John Robert Pepper | Missouri |
| Albert Ulysses Edwards | Indiana | Henry Bosworth Purl | Missouri |
| Martin Frederick Ehlers | Missouri | Harley John Riley | Missouri |
| Wil iam Walter Flora | Kansas | Noah Richard Smith | Missouri |
| Thedore Green | Illinois | Theophilus Paul Smith | Missouri |
| Samuel Wilson Harris | Missouri | John August Steinmeyer | Kansas |
| Morton David Hamisfar | Missouri | James Campbell Thompson | Missouri |
| John Frank Huntling | Nebraska | John Elias Watkins | Kansas |
| Evan D Jenkins | Kansas | Charles Delosso Weakley | Missouri |
| George Francis Kelly | Kansas | Fred Emory Webster | Kansas |
| Richard Hiram Kent | Kansas | Linsey Leonidas Wills | Missouri |

### Royal College of Dental Surgeons of Ontario.

On the 21st of April the Directors of the Royal College of Dental Surgeons had under consideration the report of the Examiners for the session just closed, and have handed out the results as follows:

The number of students registered and in attendance for the session of 1895–96 was one hundred and sixty-one.

The following passed the final examinations and received the title of L.D.S. (Licentiate of Dental Surgery):

W F Adams

R M Armstrong

T E Ball

F Britton

J J Brown

W Burnet

J M Bell

L G Campbell

H A Croll

R N Henderson

G W Hoag

O H Hutchison

J E Johnston

W E Lundy

J L Leitch

L M Mabee

F S Mercer

J F McMillan

H McQueen

C E Pearson

Percy Smith

A T Sihler

J A Simpson

W J Switzer

J G Somerville

W F Templar

W C Trotter, B.A.

Ed D Washington.

tal Surgery was conferred by Charles C. Harrison, A. M., Provost, upon the following candidates:

| NAME. | RESIDENCE. |
|---|---|
| Thomas Joseph Allen | Pennsylvania |
| William Augustus Allwood | Jamaica |
| Charles Hugh Archibald | Canada |
| George Rollin Beecher | New York |
| Hesper Beecher | Georgia |
| Edward Warnes Bohn | Pennsylvania |
| Glenni F. Bowman | Pennsylvania |
| Charles Edgar Burt | Ohio |
| James Henry Callahan | Connecticut |
| Lester Luff Carlisle | Delaware |
| George Curtis Case | Connecticut |
| Ellis Jones Chesbro | New York |
| Martin Pembroke Congdon | New York |
| Wirt Hunter Conklin | Pennsylvania |
| William Martin Copley | New York |
| Francis Correa, Jr. | Pennsylvania |
| Charles Monroe Crowell | Pennsylvania |
| Frank Hart Darragh | Pennsylvania |
| John Byers Dickinson | Arkansas |
| George William Dickson | Canada |
| Robert Anderson Dickson | Scotland |
| Arthur Gray Doane | Massachusetts |
| John Joseph Dolan | Massachusetts |
| George B. Edwards | Pennsylvania |
| Archibald C. Eglin | Pennsylvania |
| Ernest Joseph Eisen | Wisconsin |
| William Leon Ellerbeck | Utah |
| Thomas Albert Eynon | Pennsylvania |
| Frasier Milton Failing | New York |
| George Alfred Flexer | Pennsylvania |
| Charles F. Paul Groth | Pennsylvania |
| Hedley Herefoot Ham | Australia |
| Robert Dallas Hand | New Jersey |
| Frederick William Haselo | New York |
| Thomas Aloysius Herr | Massachusetts |
| Otto E. Theo. Von der Heyde | Turkey |
| Isaac Jacob Hill | Russia |
| William Charles Hoeffer | Pennsylvania |
| Powell Allan Holloway | Pennsylvania |
| Lionel Mayer Homburger | New York |
| Joseph Huggins | Washington |
| William John Ingraham | New York |
| Mack Roy Jackson | Delaware |
| Leon Samuel Joffe | Pennsylvania |
| Roy Roland Johnston | Pennsylvania |
| David Elder Kerr | Ohio |
| Frank Sydney Ketcham | New York |
| Addison Kingsbury, A. B. | Ohio |
| Harry Frisby Koontz | Ohio |
| Silas Benton Langfitt | West Virginia |
| Edward James Laughlin | Pennsylvania |
| Robert Siegfried Levy | Germany |
| Leonard Lockett | Jamaica |
| Jaime Domingo de Losada | Spain |
| William S. Louisson | Hawaiian Islands |
| Wm. H. Luffbarry, Jr. | Pennsylvania |
| Irville Olin Lyman | Pennsylvania |
| George Lawrence McAvoy | Connecticut |
| Lee Landis McDonald | Missouri |
| John Adam Mayer, Jr. | Pennsylvania |
| Evan Winter Mentzer | Pennsylvania |
| Friedrich Ernest A. Mueller | Germany |
| Frank Henry Murray | Minnesota |
| Andrew Sell Musser | Pennsylvania |
| Alfred Rodolphe Neech | Germany |
| James Pollock Nichol | Pennsylvania |
| Henry Charles O'Connor | Wisconsin |
| Nelson Thomas Paine | New York |
| George Frank Patterson | Ohio |
| Clarence Arthur Pennell | Iowa |
| Jose Perez | Cuba |
| William John Phymister | Canada |
| Charles Seymour Preston | Illinois |
| Ralph Butler Reitz, A. B. | Pennsylvania |
| Walter H. Richardson | Massachusetts |
| Eugene F. Rinebold | Pennsylvania |
| Charles Johnson Royce | Connecticut |
| Otto Carl Hermann Sawitzky | Germany |
| Robert Donkin Sayre | Canada |
| Paul Boyton Schultz | Pennsylvania |
| Henry William Shelp | Illinois |
| Edward Frank Smith | Pennsylvania |
| Fred Ethelbert Smith | Pennsylvania |
| Augustus C. St. Amand | Pennsylvania |
| Joseph Byron Stannard | Missouri |
| Adolph Stenner | Austria |
| R. A. E. Swentzell | Pennsylvania |
| Will McLain Thompson | Pennsylvania |
| Harry Drane Urian | Pennsylvania |
| Louis Lee Voigt | Pennsylvania |
| Francis William Watlington | Bermuda |
| James Thornton Watson | Pennsylvania |
| Harry Lawrence Welch | New Jersey |
| Albert D'Aran Wells | New York |
| Herbert Ely Williams | New Jersey |
| Howard Jones Williams | Pennsylvania |
| Harry Irvine Wright | Pennsylvania |
| Henry Frank Zerfing | Pennsylvania |

Total, 98.

EDWARD C. KIRK, *Dean.*

### Howard University—Dental Department.

The commencement exercises of the Dental Department of Howard University, class of 1897, were held at the Congregational Church at Washington, D. C., Monday evening, May 10, 1897. The address to the graduates was delivered by Prof.

Chas. B. Purvis, A. M., M. D. J. E. Rankin, D. D., LL. D., president of the University, also delivered an address.

The degree of Doctor of Dental Surgery was conferred upon the following graduates by J. E. Rankin, D. D., LL. D., president of the University:

| NAME | RESIDENCE. | NAME. | RESIDENCE. |
|---|---|---|---|
| Tomlin Augustus Campbell | Jamaica | Charles A. Murray | Massachusetts |
| William E. Hamilton | Texas | Keisaburo Watanabe | Japan |

Total number of Matriculates in Dental Department for the session of 1896-97, twenty-four.

### New York Dental School.

The fourth annual commencement exercises of the New York Dental School were held at the Academy of Medicine, New York, on Wednesday, May 26, 1897, at 8 o'clock P.M.

The address to the graduating class was delivered by Rev. James A. Francis.

The degree of D.D.S. was conferred on the following graduates by Professor Dwight L. Hubbard, Dean:

| NAME. | RESIDENCE. | NAME. | RESIDENCE. |
|---|---|---|---|
| J. T. T. Clark | Jamaica | Georgius H. Franzius | New York |
| William Croll | New York | James L. Gay | New York |
| Frederick A. Crookshank | New York | August F. Paulsen | New York |
| John E. Donavan | Vermont | Edward P. Whitney | Connecticut |
| Charles I. Ferris | Connecticut | August C. Wintraecken | New York |

### National University—Dental Department.

The thirteenth annual commencement exercises of the Dental Department of the National University were held, in connection with those of the Medical Department, in the National Theater, Washington, D. C., on Thursday evening, June 10, 1897.

The address to the graduating classes was delivered by Professor J. Roland Walton, D.D.S., and the valedictory address by Howard P. Cobey, D.D.S., M.D.

The degree of D.D.S. was conferred on the following graduates of the dental class:

| NAME. | RESIDENCE. | NAME. | RESIDENCE. |
|---|---|---|---|
| James J. Brennan | Pennsylvania | J. Clarence Hatton | New Jersey |
| Robert E. Buchanan | Virginia | Millard J. Holmes | New York |
| Tyrus Christman | Pennsylvania | William W. Trail | West Virginia |
| Howard P. Cobey | Maryland | Edward B. Wall | Illinois |
| William T. Heyser | Maryland | | |

### Western University of Pennsylvania—Dental Department.

The annual commencement of this Department was held in the Alvin Theatre, Phipps Building, on the 25th of March, 1897. The excercises were directed by Dr. J. C. Lange, Dean of the Medical Faculty, and were opened with prayer by the Rev. E. Re Donehoo. The opening address was made by J. C. White, president of the Board of Trustees. A very interesting address was also delivered by Chancellor W. J. Holland. The roll of candidates for the degree of D.D.S. was called by the Dean Dr. J. G. Templeton, when the following received the degree:

| | | |
|---|---|---|
| Henry Redmond | Nevin Harbaugh Snyder | George Leon McFarland |
| Adolph T. Bowers | John Howard Sloan | |

### Boston Dental College.

The thirtieth annual commencement exercises of the Boston Dental College were held in the Berkeley Temple, Boston, Mass., on Wednesday evening, June 16, 1897.

The annual address was delivered by Rev. Stephen N. Roblin, and the valedictory by Seth B. Hilborn, D.D.S.

The degree of D.D.S. was conferred on the following graduates by I. J. Wetherbee, D.D.S., President of the college:

| NAME. | RESIDENCE. | NAME. | RESIDENCE. |
|---|---|---|---|
| George H. Akins | Massachusetts | Calvin N. Lassonde | Vermont |
| Harry J. Baker | New Hampshire | Benjamin Lewis | Vermont |
| Daniel J. Barnicle | Massachusetts | Jos. E. Livingston | Vermont |
| Alston F. Barrett | New Hampshire | Sverker Luttropp | Vermont |
| Joseph A. Barrett | Massachusetts | Geo. E. Maguire | Vermont |
| Daniel W. Bradt | Massachusetts | Joseph Massel | Vermont |
| Herbert R. Brunton | Massachusetts | E. M. Montgomery | New Burnswick |
| John F. Caulfield. | Massachusetts | Joanna R. Nahrung | Massachusetts |
| Marie H. F. Caron | Massachusetts | Wm. B. Osgood | Massachusetts |
| William A. Cleary | Massachusetts | Joseph H. Peront | Massachusetts |
| Neal F. Clifford | Massachusetts | Percival Phillips | Massachusetts |
| Edward E. Copeland | Massachusetts | Robert A. Pierce | Massachusetts |
| John S. Cunard. | Massachusetts | John P. Rattigan | Massachusetts |
| John Denby | Rhode Island | Russell A. Richards | Massachusetts |
| James B. Duffy | New Hampshire | Henry S. Robinson | Rhode Island |
| Edward M. Ennis | New Hampshire | James A. Scanlan | Rhode Island |
| Raym'd M. Gaylord | Massachusetts | Jacob Schwartz | Rhode Island |
| Thomas H. Gifford | New Hampshire | Isaac H. Small | Rhode Island |
| Andrew F. Hall | Massachusetts | Fred'k B. Stevens | Rhode Island |
| Seth B. Hilborn | Maine | Emil Strohn | Rhode Island |
| James D. Hogan | Nova Scotia | Samuel H. Sweatt | Rhode Island |
| Fred'k A. Holden | Massachusetts | W. C. Thompson | Rhode Island |
| Hiland F. Holt | New Hampshire | Stanley Towle | New Hampshire |
| Sara P. Hooker | Massachusetts | Jerome P. Tripp | New Hampshire |
| Anthnay V. Hoppe | Massachusetts | Arie L. Tscherniatovski | Russia |
| John F. Howland | Massachusetts | Arthur F. Weaver | Massachusetts |
| Ervin A. Johnson | Vermont | Truman R. Wilbur | Massachusetts |
| Orion Kelley | Vermont | Lewis W. Wilson | Massachusetts |
| William J. Kelley | Vermont | | |

### Columbian University—Dental Department.

The commencement at this college was held Tuesday, May 4th, 1897. Service opened with a prayer by Rev. Joseph T. Kelly. An address to graduates was given by Prof. J. Hall Lewis. The valedictory was delivered by E. H. Coumbe, D. D. S. Award of prizes by Prof. H. C. Thompson.

The degree of D.D.S. was conferred by President B. L. Whitman, D. D., upon the following candidates:

| NAME. | RESIDENCE. | NAME. | RESIDENCE. |
|---|---|---|---|
| Wm. F. Ankeney | Maryland | James G. Haskell | Kentucky |
| Damon A. Binkert | Illinois | Frank H. Waite, Ph. D | Maryland |
| Emmett M. Carter | District of Columbia | Howard A. Wiltberger | Pennsylvania |
| Eppa H. Coumbe | Virginia | Richard Washington, M. D | Virginia |
| T. B. Cochran | Virginia | Elmer F. Yount | District of Columbia |
| George E. Hurley | Massachusetts | | |

### Western Dental College.

The annual commencement of this institute was held at Coates' Opera House, Kansas City, Mo.

Invocation by Rev. W. P. George, D. D. The annual address was delivered by Rev. Geo. H. Combs. Faculty address by Geo. Halley, M. D. Mr. Harry Lightner of the class of '97 gave the valedictory.

The degree of D. D. S. was conferred by D. J. McMillan, M. D., D. D. S., upon the following candidates:

| | | |
|---|---|---|
| Robert C. Anderson | Robert Hunter | David Arguyle Rouner |
| Thomas Wesley Arnold | Charles W. Keel | Will Strouse Richardson |
| Otto Lawrence Barrett | Louis Kretzler | Dot Swope Sands |
| Oscar Bland | Louis Fielding Lamkin | Albert Martin Sears |
| Edwin James Brown | Harry Llewellyn Lightner | Joseph Reed Shannon |
| Edward E. Carpenter | Samuel Clinton Marshall | Munroe Swofford |
| Thomas William Carter | John J. Means | Lutie Florence Smith |
| Charles Milton Cheney | Frank D. McMillen | Edward F. K. Sparks |
| Mary Eliza Downing | Charles Other Metzler | Augustus J. Thompson |
| Thomas Summers Dean | Fred W. Miller | Leslie Asberry Vandiver |
| Gus H. Ehlers | John Herbert Moran | Bert Winfield Vickery |
| Thomas Benton Fleming | Edgar Allen Morris | Marion Warner |
| Lloyd Hamill | Arthur Leroy Murphy | John Thomas Watters |
| Samuel Marion Hamby | Orin Harry Peak | George Edward Wheeler |
| John Dott Harper | D. T. Polk | Joseph K. Will |
| Harry Van Hart | Elmer E. Reed, M. D. | Martin Luther Williams |
| J. Amstead Harlan | James Ira Richardson | Clarence Milford Wilson |
| John Howard | | |

### Atlanta Dental College.

This Institution celebrated its fourth annual commencement, March 24th, 1897, in the Auditorium of the Grand Opera House.

An address was delivered by Dr. Wm. Crenshaw, Dean of the Faculty, in which he reviewed the work of the past year, characterizing it as the most successful in the history of the college. The annual oration was delivered by Prof. Chas. W. Lane. Dr. C. E. Melbourne delivered the valedictory.

After these addresses the degree of D.D.S. was conferred upon the following candidates:

| NAME. | RESIDENCE. |
|---|---|
| T. H. Abbey | Georgia |
| A. D. Adair | Alabama |
| T. A. Allen | North Carolina |
| A. W. Askew | Georgia |
| E. W. Bethea | Georgia |
| B. B. Brooks | Georgia |
| C. H. Brown | Tennessee |
| W. N. Brown | Georgia |
| J. T. Burns | Alabama |
| R. L. Carllautt. | Louisiana |
| W. H. Chambers | Alabama |
| C. N. Clark. | Florida |
| J. Q. Clifton | Georgia |
| J. O. Collar | Georgia |
| G. W. Cooper | Georgia |
| E. A. Crawford | North Carolina |
| C. W. Dedge | Georgia |
| J. A. Dickson | South Carolina |
| J. A. DuPre | Tennessee |
| S. F. Ellis | Georgia |
| E. R. Eversburg | Texas |
| W. E, Floyd | Alabama |
| J. P. Greer | Georgia |
| L. C. Halzendorf | Georgia |
| C. T. Hawes | North Carolina |
| E. L. Holmes | Georgia |
| Fred Johnson | Virginia |
| F. F. Johnson | South Carolina |
| Jessee Jones | Virginia |
| S. B. Klutz. | North Carolina |
| C. D. Livingston | North Carolina |
| J. T. McCracken | North Carolina |
| L. F. McKey | Georgia |
| A. E. Merritt | Georgia |
| C. R. Mitchell | Alabama |
| H. W. Neal | Georgia |
| H. A. Norrell | Georgia |
| W. W. Palmer. | Tennessee |
| R. A. Rozier | North Carolina |
| C. C. Smith | Indiana |
| C. W. Spence | Georgia |
| G. W. Staples | Texas |
| George H. Tate | Mississippi |
| M. E. Turner | Florida |
| F. B. Walton | Georgia |
| B. N. Webb | Mississippi |
| William Weibusch | Texas |
| E. C. Wellborn | Georgia |
| A. J. Williams | Georgia |
| W. P. Wright | Mississippi |

### Vanderbilt University.

The commencement of the Department of Dentistry, Vanderbilt University, Nashville, Tenn., having 170 students registered the past session assured on the evening of April 1st, at the Vondome Theater in this city.

The address on the part of the class was delivered by S. W. Brand, D.D.S. of Illinois, that on the part of the Faculty by the Rt. Rev. Bishop Gailon of the Episcopal Church.

Forty-two candidates received the degree of Doctor of Dental Surgery, conferred by the Chancellor Jas. H. Kirkland, A.M Ph. D.

| NAME. | RESIDENCE. |
|---|---|
| D. A. Babcock | Illinois |
| E. H. Barker | Kentucky |
| P. W. Beckham | Virginia |
| H. P. Birdsong | Mississippi |
| A. A. Brand | Illinois |
| L. W. Brand | Illinois |
| E. B. Cade | Tennessee |
| M. L. Chatham | Tennessee |
| C. C. Cooke | Texas |
| Vassar Crenshaw | Alabama |

| NAME. | RESIDENCE. | NAME. | RESIDENCE. |
|---|---|---|---|
| W. L. Dazey | Texas | W. T. McDaniel | Alabama |
| J. F. Dismukes | Kentucky | J. D. McKenney | Indiana |
| H. S. Doolan | Kentucky | A. T. McMillin | Arkansas |
| H. H. Glover | Georgia | C. W. MeGuiar | Kentucky |
| W. H. Golson | Alabama | J. W. Neblett | Tennessee |
| E. F. Griffin | Mississippi | R. H. Perry | Kentucky |
| J. T. Hays | Tennessee | E. T. Phillips | Kentucky |
| R. E. Hewes | Louisiana | J. A. Richards | Louisiana |
| J. H. Hogan | Illinois | E. J. Snider | South Carolina |
| J. C. Howard | Kentucky | J. H. Pence | Mississippi |
| W. J. Hunt | Tennessee | C. H. Tandy | Kentucky |
| E. M. Hunter | Tennessee | F. I. Tarrant | Alabama |
| R. E. D. Irvin | Alabama | C. S. Taylor | Tennessee |
| G. N. Keeling | Tennessee | J. C. Walker | South Carolina |
| J. O. Merrill | Tennessee | R. E. Ware | North Carolina |
| W. H. Moore | Kentucky | D. T. Woodard | Tennessee |

### University of Michigan.

The annual commencement of the University of Michigan was held July 1st, at University Hall, Ann Arbor. The exercises were opened with prayer. The commencement oration was delivered by Andrew Sloan Draper, L.L.D., President of the University of Illinois.

The degree of D.D.S. was conferred by Dr. James B. Angell, L.L.D., President of the University, upon the following candidates :

| | | |
|---|---|---|
| William Henry Baker | Harry Sanburn Holmes | Albert Jesse Reed |
| Elmer Isaac Beistle | Samuel Wesley Honey | Dessie Brown Robertson |
| James Carroll Blair | Fred Holloway Hood | John Milton Rosenthal |
| June Alice Burr | Frank Ward Hewlett | Samuel Kane Scharlott |
| Thomas Ed. Carmody | Samuel William Hussey | Arthur Walker Schurtz |
| Herbert T. Cummings | Wendell Howard Johnson | Charles Elsworth Sheldon |
| Guy Henry Dennis | Frederick William Joslin | Charles Lindsley Sitzer |
| Albert Julius DuBois | Frederick John Klein | Luman Reed Slawson |
| Arthur Benton Dutch | Gustavus Eugene Kuhl | Charles Clifford Stone |
| George Daniel Edgar | Frank Dwight Loomis | Delmer Willis Stoup |
| Henry Christopher Fiebig | Kennith McKay | Edward Leonard Vaile |
| Frank Russell Fletcher | Frank T. McNamara | James Norman Vodrey jr. |
| John E. Graham | Roland S. Mitchell | Harry Douglas Watson |
| Selwyn Sumner Greeley | Blaine Bowman Pettit | Albert Joseph Wildanger |
| Albert Benjamin Green | William Racine Purmort | Albert John Wolfert |
| Grant Simon Hadley | Carlos Walter Putt | |
| Louis Richards Hoelzle | Oloff Wellington Randall | |

There were present in the Dental Department during the year just closed, in all, 199 students.

### Cincinnati College of Dental Surgery.

The Cincinnati College of Dental Surgery, Cincinnati, Ohio, held its annual commencement exercises on April 2nd, in the Auditorium "Odd Fellows' Temple," cor 7th and Elm Streets.

The degree of Doctor of Dental Surgery was conferred upon the following gentlemen:

| | | |
|---|---|---|
| John Peter Becker | Hiram Benson Hill | John Downing Scott |
| Joseph Edward Bruchie | William Henry Hill | Leonard Clark Shaw |
| Lewis E. Davis | Charles R. Lawrence | Stephen Girard Siebern |
| Herschel P. Ferris | Roy G. Merriman | Chas. Stanley Smith |
| William O. Gillham | Louis C. O'Donnell | Walter Powell Stewart |
| Edmund S. Grant, Jr. | Charles A. Pfafflin, M.D. | Roland S. Van Hise |
| Newell Henry Grove | Robert M. Prather | Robert G. Wiggers |
| Courtney Stickney Haver | Charles Arthur Row | Martin L. Williamson |
| Claude Thos. Hickman | Edward Lamport Schell | Christ. Miller Zeller |

The number of the matriculants for the session was 76.

Addresses appropriate for the occasion were delivered by the Hon. Francis B. James, President Board of Trustees; Dr. G. S. Junkerman, Dean of the Faculty; Hon. Levi C. Goodale. Class oration was delivered by Dr. J. E. Bruchie. Almuni, Graduates, Board of Trustees and Faculty enjoyed after the exercises a banquet at the Grand Hotel of many courses including speeches and music.

### Northwestern University Dental School.

The commencement exercises of this college were held in the Grand Opera House, Chicago, April 2d, 1897, at 2 P. M.

Invocation by Rev. W. H. Holmes. Salutatory Address, Otto Wolfrum. Annual Report and Mandamus by Dr. Theo. Menges, B. S., D. D. S. Doctorate Address, Rev. Willard Scott. Valedictory, Sam'l Straith, D. D. S.

The degree of D. D. S. was conferred by Henry Wade Rogers, L. L. D., President of the University, upon the following candidates:

| NAME. | RESIDENCE. | NAME. | RESIDENCE. |
|---|---|---|---|
| Algernon Alfonso Atwood | Illinois | Walter Cone Beesley | Illinois |
| George Elmer Ames | Illinois | Newton E. Ballou | Michigan |
| Frank T. Armstrong | Iowa | Marcia Aalowine Berlyn | Indiana |
| William Orwin Allen | Montana | Bert Hamilton Biglow | Wisconsin |
| Edward Francis Burns | Wisconsin | Charles James Breckenridge | Illinois |
| James Vining Brown | Ontario | David Shaffer Brogunier | Illinois |
| William Henry Harrison Barker | South Dakota | Nathan Lewis Burke | Michigan |
| Frank Allen Bryant | Illinois | Rudolph Bostelman | Illinois |
| Rudulph Heinrich Bloese | Illinois | Herbert Elliott Burnett | Montana |
| William Beidler | Illinois | Jacob J. Bing | Wisconsin |
| Lewis Taber Bristol | Illinois | Frederick Joseph Brown | Iowa |
| Ira Clifton Brownlie | Iowa | Nellie Babcock | Indiana |
| Harold Ingraham Bragg | Missouri | James Leonard Babcock | Indiana |
| Alvin William Barthel | Illinois | Leopold Bertrand | Louisiana |
| George Isaac Bevier | Iowa | Charles Walker Chubbuck | Missouri |
| Albert Earnest Brandes | Wisconsin | Clarence Eugene Coy | Illinois |
| | | Walter Henry Caradine | Wisconsin |

| NAME. | RESIDENCE. |
|---|---|
| Samuel Hamilton Chase, | Wisconsin |
| Winfield Scott Clark | Illinois |
| Roy Arthur Case | Iowa |
| Emerson E. Cherrington | Illinois |
| Felix Bucknam Cassidy | Maryland |
| Louis Edwin Crandall | Illinois |
| John Darwin Davis | Illinois |
| John Thomas Dixon | Minnesota |
| Alexander Clifford Dawson | Ontario |
| Edward Henry Drews | Wisconsin |
| Wilbur M. Dace | Illinois |
| Albert George Doepp | Illinois |
| Albert James De Velde | Illinois |
| Samuel Leonard Drake | Iowa |
| James William Erringer | Illinois |
| Thomas Sidney Eldredge | Illinois |
| William Ebenezer Ellis | Nebraska |
| Elkan Washington Fishell | Illinois |
| Harry Maynard Fuller | Illinois |
| Charles Frederick Freiberg | Illinois |
| Louis Elsworth Funk | Illinois |
| G. Walter Gray | Missouri |
| John Dawson Griffin | Illinois |
| Elmond Charles Gates | Australia |
| William Morse Griswold | Minnesota |
| George Wellington Gage | Iowa |
| Walter Wilson Griffith | Indiana |
| Harry Francis Holder | Illinois |
| Charles Prescott Herbert | Illinois |
| Gordon Ransom Hickey | Wisconsin |
| Otto Charles Heine | Illinois |
| Joseph Milton Hamilton | Wisconsin |
| John Richard Hanley | Illinois |
| James Archer Harper | Illinois |
| William Joseph Hacking | Manitoba |
| William James | Wisconsin |
| John A. Jones | Wisconsin |
| Therese Kass | Illinois |
| David Alexander Kennedy | Ontario |
| Charles Willard Kramer | Pennsylvania |
| Herbert C. Kettell | Iowa |
| William Nicholas Klumb | Indiana |
| William Woodsworth Kenney | Ontario |
| Christine Margaret Konantz | Illinois |
| Herman Michael Karsten | Iowa |
| Otto Ulysses King | Indiana |
| Elmer Anthony Lentz | South Dakota |
| Elmer Edwin Lampert | Illinois |
| George Buchanan McFarlane | Illinois |
| Carl Wilson McGaughey | Illinois |
| George Bester McKinney | Illinois |
| Elwood Joseph Miller | Illinois |
| Walter Garner Morris | Oregon |
| Emma Frances Macdonald | Illinois |
| Isaac Perkins Meredith | Missouri |
| Melvin Edgar Merker | Illinois |
| Edward James Martin | Illinois |
| William Howard Overmeyer | Illinois |
| Joseph Oettinger | Indiana |
| Elmer Eugene Prescott | Minnesota |
| Frederick McDonald Rawlings, | Illinois |
| James H. Ridgeway | Wisconsin |
| David Phillip Redemann | Iowa |
| Richard Rogers | Illinois |
| Jesse William Ritter | Michigan |
| Elwood Emory Ross | Illinois |
| Joseph Clarence Robinson | Iowa |
| Harry David Shaner Reisinger, | Indiana |
| William Nixon Ratliff | Indiana |
| Edward Piere Skinner | Illinois |
| Alice Steeves | Canada |
| John Schwartz | Iowa |
| George Albion Stewart | Iowa |
| James Walter Shaffer | Illinois |
| Minna Sanner | Germany |
| Samuel Straith | Michigan |
| William Preston Scott | Michigan |
| Elam Alden Sanderson | Kentucky |
| Isaac Sundberg | Iowa |
| David Spar Stiver | Illinois |
| Albert Edward Swanson | Iowa |
| Arthur Burton Simpkins | Illinois |
| Herbert Haze Stevens | Iowa |
| Carl Erick William Skogsborg, | Sweden |
| Charles Albert Taylor | Iowa |
| William Henry Thrower | Iowa |
| George Francis Tibbits | Illinois |
| James X. Thompson | North Dakota |
| Stephen Edgar Thompson | Indiana |
| William Van Hon | Iowa |
| Otto Wolfrum | Wisconsin |
| Albert Elsworth Wrixon | Illinois |
| Levi Grant Woodruff | Iowa |
| James Madison Weems | Texas |
| Samuel Flagler Walton | Illinois |
| Eugene Edgar Willsey | Illinois |

